# Serum 25(OH)D Levels and Dental Caries Experience in Children: A Cross-Sectional Study

**DOI:** 10.3290/j.ohpd.c_2779

**Published:** 2026-08-13

**Authors:** Çiğdem Güler, Emine Yurdakul Ertürk, Didem Odabaşı, Emre Serhan Alper

**Affiliations:** a Çiğdem Güler Professor, Department of Pediatric Dentistry, Faculty of Dentistry, Ordu University, Ordu 52200, Turkey; conceived and designed the study, contributed to the methodology, formal analysis, and data interpretation, supervised the study and was responsible for project administration, and reviewed, revised, and approved the final manuscript; b Emine Yurdakul Ertürk Associate Professor, Department of Pediatrics, Ordu University Training and Research Hospital, Ordu 52200, Turkey; conceived and designed the study, contributed to the methodology, formal analysis, and data interpretation, and reviewed, revised, and approved the final manuscript; c Didem Odabaşı Assistant Professor, Department of Pediatric Dentistry, Faculty of Dentistry, Ordu University, Ordu 52200, Turkey; conceived and designed the study, contributed to the methodology, formal analysis, and data interpretation, and reviewed, revised, and approved the final manuscript; d Emre Serhan Alper Research Assistant, Department of Pediatric Dentistry, Faculty of Dentistry, Ordu University, Ordu 52200, Turkey; conceived and designed the study, conducted the clinical investigation, performed data collection, and prepared the original draft of the manuscript, and reviewed, revised, and approved the final manuscript

**Keywords:** children, cross-sectional study, dental caries, vitamin D

## Abstract

**Purpose:**

The relationship between serum 25-hydroxyvitamin D [25(OH)D] levels and dental caries in children remains controversial. This study aimed to investigate the relationship between current serum 25(OH)D levels and dental caries experience in children.

**Materials and methods:**

This cross-sectional study included children aged 3 to 15 years without systemic disease who presented to the Department of Pediatrics, Ordu University Training and Research Hospital, Ordu, Turkey, between November and December 2023. Serum 25(OH)D levels obtained from routine blood tests and intraoral examinations performed on the same day were evaluated retrospectively. Dental caries experience was assessed using dmft and DMFT indices according to World Health Organization (WHO) criteria. Statistical analyses were performed to evaluate the relationship between serum 25(OH)D levels and caries experience.

**Results:**

A total of 326 children were included (172 girls, 52.8%; 154 boys, 47.2%). The mean serum 25(OH)D level was 50.08 ± 16.68 nmol/l, and the mean combined caries index (decayed, missing, and filled primary teeth [dmft] and decayed, missing, and filled permanent teeth [DMFT]) was 3.66 ± 3.44. No statistically significant relationship was observed between serum 25(OH)D levels and caries experience (*P* > 0.05). Girls had statistically significantly lower serum 25(OH)D levels than boys (46.03 ± 15.21 nmol/l vs. 54.63 ± 17.10 nmol/l, *P* < 0.001).

**Conclusions:**

Within the limitations of this cross-sectional study, no statistically significantly relationship was found between current serum 25(OH)D levels and dental caries experience in children. Further longitudinal studies with comprehensive assessment of potential confounding factors are needed to better clarify this relationship.

Dental caries is one of the world’s most common chronic diseases and primarily affects children.^22,44
^ Although it is preventable in childhood, high prevalence rates of early childhood caries have been reported in many populations.^4^ Several factors contribute to the development of dental caries, including a combination of physical, biological, sociodemographic, and behavioural elements.^20,36
^


Minerals and vitamins play a crucial role in the formation and maintenance of tooth structure. Among these nutrients, vitamin D is essential for calcium–phosphate homeostasis, bone metabolism, and immune function.^18,41
^ Vitamin D is a fat-soluble steroid hormone that influences the small intestine, kidneys, bone, and muscle tissues. Polymorphisms in vitamin D receptor genes have also been associated with oral health conditions such as dental caries and periodontal disease.^12,18,33
^


Vitamin D deficiency is a widespread public health concern across both high-latitude and sunny countries.^41,45
^ Although deficiency is common in northern regions such as Canada and Northern Europe, studies conducted in Brazil, a country with abundant sunlight, have shown that serum vitamin D levels may still be insufficient due to lifestyle changes and sun-avoidance behaviours.^38,41
^


Vitamin D has been suggested to influence the developing dentition primarily through its biologically active form, 1,25-dihydroxyvitamin D [1,25(OH)_2_D]. This metabolite maintains calcium and phosphorus balance and may contribute to the mineralisation of dental hard tissue.^6,18
^ Previous studies have suggested that vitamin D may be involved in enamel development and the functional activity of ameloblasts and odontoblasts.^6,13
^


Low serum 25(OH)D levels have been associated with various oral conditions, including enamel hypoplasia and developmental enamel defects, although findings remain inconsistent.^11,13,37
^ They may also impair remineralisation by altering salivary calcium and phosphate concentrations and weaken immune defence mechanisms, as vitamin D is essential for the synthesis of antimicrobial salivary peptides.^13,18
^


Serum 25(OH)D is widely used as a biomarker of current vitamin D status.^25^ However, evidence regarding the relationship between serum 25(OH)D levels and dental caries remains inconsistent. Several studies have reported an inverse association between vitamin D and caries risk,^8,15,28
^ whereas others have shown weak or non-significant associations.^11,15
^ A study conducted in Portugal reported an association in permanent teeth of 7-year-old children,^9^ while a study carried out in Qatar demonstrated a high frequency of vitamin D deficiency among children with caries.^5^ In Turkey, studies have reported widespread vitamin D deficiency among children but no significant association between 25(OH)D levels and dental caries or molar incisor hypomineralisation (MIH).^1,10,24
^ Prospective cohort studies and nationally representative samples have also examined this relationship, providing important evidence on whether vitamin D status may predict future caries outcomes.^11,15,31,35
^


Given these conflicting findings, further research is required to clarify the relationship between serum 25(OH)D levels and dental caries in children. This study aimed to investigate the relationship between current serum 25(OH)D levels and dental caries experience in children.

## Materials and methods

### Study Design and Sample Size

This was a cross-sectional observational study. No a priori sample size calculation was performed; the sample consisted of all eligible children who presented during the study period. This is acknowledged as a limitation, particularly regarding analyses involving MIH and enamel hypoplasia, for which larger sample sizes are typically required to detect modest associations.

### Inclusion Criteria

The inclusion criteria were as follows:

paediatric patients aged 3 to 15 years;children requiring routine blood tests;no prior diagnosis of systemic disease;no history of radiotherapy, chemotherapy, hormone therapy, or vitamin D supplementation within the past 6 months;parents/guardians who read and voluntarily signed the informed consent form prepared for the study.

### Exclusion Criteria

The exclusion criteria were as follows:

children younger than 3 years or older than 15 years;children who did not require routine blood tests;children with systemic disease;children who had undergone radiotherapy, chemotherapy, or hormone therapy, or taken vitamin D supplements within the past 6 months;parents/guardians who did not agree to provide informed consent.

### Clinical Examination and Calibration

Each child underwent an intraoral examination on the same day that a venous blood sample was drawn. Examinations were conducted by a paediatric dentistry research assistant. The examiner underwent a calibration process that included theoretical training on World Health Organization (WHO) criteria for dental caries, MIH, and enamel hypoplasia; clinical training on 20 children not included in the study; and assessment of intraexaminer reliability, yielding a Kappa score of 0.87 for caries detection and 0.82 for MIH diagnosis, indicating substantial agreement.

A structured data collection form was used; no personal identifiers were recorded except age and sex (biological sex: male/female).

### Dental Caries Assessment

Caries status was recorded separately for decayed, missing, and filled teeth in primary dentition (dmft) and decayed, missing, and filled teeth in permanent dentition (DMFT) using WHO criteria (cavitated lesions only); dmft scores were recorded for children in primary dentition (≤ 6 years), whereas dmft and DMFT scores were used for children in mixed dentition and DMFT scores for those in permanent dentition (> 6 years).^43^ Because the sample covered a wide age range (3 to 15 years) and included many children in mixed dentition, dmft and DMFT distributions were heterogeneous across age groups. For this reason, combined caries experience (dmft + DMFT) was used for analyses of overall caries experience. The combined caries experience was classified as:

0 caries = no caries;1 to 3 caries = low caries experience;> 3 caries = high caries experience.

This approach has been adopted in previous epidemiological studies involving children, particularly to facilitate interpretation of overall caries burden in mixed dentition populations.^26,39,40
^


The presence of enamel hypoplasia, MIH, and occlusal relationships were also recorded. MIH diagnosis followed the criteria of Wetzel and Reckel.^42^ Teeth presenting with enamel hypoplasia were excluded from dmft/DMFT calculations to avoid potential overestimation of caries-related defects.

### Assessment of Serum 25(OH)D

Serum 25(OH)D values were classified by a paediatrician. For consistency with international reporting standards, vitamin D levels were converted to nmol/l (1 ng/ml = 2.5 nmol/l) and categorised as follows:^19^


< 50 nmol/l: deficiency;50 to 75 nmol/l: insufficiency;> 75 nmol/l: sufficiency.

### Statistical Analysis

Statistical analyses were conducted using SPSS version 25 (IBM, Armonk, NY, USA). Categorical variables were presented as frequencies and percentages, and numerical variables as mean, standard deviation, and minimum and maximum values. Normality was evaluated using a Kolmogorov-Smirnov test.

Because caries variables were not normally distributed, a Spearman rho was used to assess correlations. Group comparisons were performed using a Kruskal-Wallis test for non-normally distributed numerical variables across multiple groups and Mann-Whitney U or *t* tests where appropriate for two-group comparisons.

Given the small number of MIH and enamel hypoplasia cases, only descriptive statistics were reported for these conditions, as the study was underpowered to explore associations with vitamin D. A post-hoc detectable effect size assessment was performed to estimate the minimum correlation coefficient detectable with 80% statistical power at a significance level of α = 0.05. *P* < 0.05 was considered statistically significant.

This cross-sectional observational study was conducted in accordance with the principles of the Declaration of Helsinki. Ethical approval was obtained from the Ordu University Clinical Research Ethics Committee (approval no: 2023/295). The study was carried out between 20 November and 21 December 2023 among paediatric patients admitted to the Department of Pediatrics, Ordu University Training and Research Hospital. Informed consent was obtained from parents or legal guardians of all participants, and age-appropriate assent was obtained from the children. The null hypothesis was that no significant relationship exists between current serum 25(OH)D levels and caries experience in children.

## Results

A total of 326 children were included in the study, of whom 172 (52.8%) were female and 154 (47.2%) were male. The mean age was 8.95 ± 3.52 years.

### Serum 25(OH)D Levels 

The mean serum 25(OH)D level was 50.08 ± 16.68 nmol/l (range 14.75 to 98.75 nmol/l) after conversion from ng/ml.

Vitamin D categories were as follows: deficiency (< 50 nmol/l), 172 children (52.8%); insufficiency (50 to 75 nmol/l), 118 children (36.2%); and sufficiency (> 75 nmol/l), 36 children (11.0%).

There was a statistically significant difference in serum 25(OH)D levels by sex (*P* < 0.001), with girls exhibiting lower levels than boys (46.03 ± 15.21 nmol/l vs. 54.63 ± 17.10 nmol/l, respectively). Participant characteristics and serum 25(OH)D levels are shown in Table 1.

**Table 1 table1:** Participant characteristics and serum 25(OH)D levels

Variable		n (%) or mean ± SD
Total number of participants		326
Age, y		8.95 ± 3.52
Sex	Female (n, %)	172 (52.8%)
	Male (n, %)	154 (47.2%)
Serum 25(OH)D (nmol/l)		50.08 ± 16.68 (range 14.75–98.75)
Serum 25(OH)D categories (n, %)	Deficiency (< 50 nmol/l)	172 (52.8%)
	Insufficiency (50–75 nmol/l)	118 (36.2%)
	Sufficiency (> 75 nmol/l)	36 (11.0%)


### Caries Experience by Dentition Stage

Caries experience was evaluated separately for primary and mixed or permanent dentitions and as a combined measure:

dmft (≤ 6 years, n = 90): 1.00 ± 1.11 (range 0 to 3);dmft + DMFT or DMFT (> 6 years, n = 236): 4.68 ± 3.48 (range 0 to 16);combined dmft + DMFT (n = 326): 3.66 ± 3.44 (range 0 to 16).

Combined caries experience was categorised as:

0 caries: 76 (23.3%);1 to 3 caries: 113 (34.7%); > 3 caries: 137 (42.0%).

Combined caries distribution by category is shown in Table 2. Given the wide age range and unequal distribution between dentition stages, analyses were performed in the total study population using overall caries experience measures.

**Table 2 table2:** Caries indices by dentition stage and combined

Group		n	Mean ± SD	Minimum–maximum

Primary dentition (≤ 6 y), dmft		90	1.00 ± 1.11	0–3
Mixed or permanent dentition (> 6 y), dmft + DMFT or DMFT		236	4.68 ± 3.48	0–16
Combined caries experience (dmft + DMFT)		326	3.66 ± 3.44	0–16
Combined caries categories (n,%)	0 (no caries)	76 (23.3%)		
	1–3 caries (low caries experience)	113 (34.7%)		
	> 3 caries (high caries experience)	137 (42.0%)		


### Relationship between Serum 25(OH)D and Caries Experience

Mean serum 25(OH)D levels by caries category were:

0 caries: 49.90 ± 17.33 nmol/l;1 to 3 caries: 49.38 ± 15.70 nmol/l; > 3 caries: 50.78 ± 17.15 nmol/l.

The distribution of mean serum 25(OH)D levels by caries category is shown in Table 3. There was no statistically significant difference in serum 25(OH)D levels across caries categories (Kruskal-Wallis *P* = 0.896).

**Table 3 Table3:** Serum 25(OH)D (nmol/l) by caries category

Caries category	n	25(OH)D, mean ± SD	*P* value
0 (no caries)	76	49.90 ± 17.33	0.896
1–3 caries (low caries experience)	113	49.38 ± 15.70	
> 3 caries (high caries experience)	137	50.78 ± 17.15	


Spearman correlation analysis demonstrated no significant relationship between serum 25(OH)D levels and total caries experience (*P* = 0.746).

### MIH, Enamel Hypoplasia (Reported Descriptively Due to Very Small Sample Size)

Because the study was underpowered for MIH (n = 2) and enamel hypoplasia (n = 5), MIH was present in only two children (0.6%), both with insufficient vitamin D levels, and enamel hypoplasia was present in five children (1.5%), with two in the deficient and three in the insufficient vitamin D range.

### Occlusal Relationships

Occlusal classifications were as follows:

Class I: 237 (72.7%);Class II: 63 (19.3%);Class III: 26 (8.0%).

There was no statistically significant association between serum 25(OH)D levels and occlusal relationships (*P* = 0.296).

## Discussion

The aim of this cross-sectional study was to investigate the relationship between current serum 25(OH)D levels and dental caries in a paediatric population. Although previous studies conducted in Turkey and other countries have reported high rates of vitamin D deficiency in children, the literature remains inconsistent regarding its relationship with dental caries.

In this study, vitamin D deficiency and insufficiency were highly prevalent. The mean serum 25(OH)D level was 50.08 ± 16.68 nmol/l. Only 11% of children achieved sufficient levels, and more than half of the participants were classified as vitamin D deficient. Similar prevalence rates have been reported in other regions of Turkey, supporting the notion that vitamin D deficiency is an important public health issue in Turkish children.^1^


The study period (November to December) coincided with months of limited sunlight exposure; however, low vitamin D levels have also been widely reported in countries with abundant sunshine, such as Brazil and Saudi Arabia.^2,38
^ This suggests that vitamin D status cannot be attributed solely to geographic sunlight exposure but is also influenced by dietary patterns, cultural practices, clothing style, lifestyle, time spent indoors, and environmental factors. However, the present study did not collect data on these covariates; therefore, interpretations relating to sunlight exposure, diet, and lifestyle should be considered cautiously.

When serum 25(OH)D levels were analysed by sex, girls had significantly lower levels (46.03 ± 15.21 nmol/l) than boys (54.63 ± 17.10 nmol/l). This result is consistent with studies conducted in both Turkey and Bahrain.^21,23
^ Although clothing-related sun exposure differences may play a role,^16^ the persistence of this sex difference even in countries without traditional clothing norms suggests that biological and behavioural factors such as dietary habits, time spent outdoors, and hormonal influences may also contribute.^17,29,30,34
^ However, because data on sun exposure habits and clothing practices were not collected in the present study, these interpretations should be considered speculative.

Consistent with previous studies in Turkey reporting high caries prevalence (69.2% to 82.6%),^3,14,32
^ the mean combined dmft/DMFT value was elevated, and 42.0% of participants were classified in the ‘> 3 caries’ group. Caries risk is multifactorial and influenced by oral hygiene, diet, socioeconomic status, developmental defects, and potentially vitamin D deficiency. Because the current study did not collect or adjust for potential confounders, such as dietary vitamin D intake, sunlight exposure, oral hygiene behaviours, or socioeconomic variables, the analysis reflects only crude associations.

In the present study, no statistically significant association was found between serum 25(OH)D levels and dental caries. Although many studies report both significant and nonsignificant associations, the general trend in the literature suggests an inverse association between vitamin D levels and caries.^7^ Large cohort and nationally representative studies such as those conducted by Gyll et al,^15^ Dudding et al,^11^ and Navarro et al^31^ reported mixed results, further demonstrating inconsistency in the current evidence. The lack of a significant association in the present study should be interpreted in light of the cross-sectional design, the use of a single-time serum 25(OH)D measurement reflecting current vitamin D status, the cumulative nature of dental caries, the multifactorial aetiology of caries, and the limited control of potential confounding factors.

A post-hoc detectable effect size assessment indicated that, with a sample size of 326 and α = 0.05, the study had 80% power to detect correlations of approximately r = 0.15 or greater. Therefore, the absence of a statistically significant association is unlikely to be explained solely by sample size limitations, although weaker associations may have remained undetected.

Enamel hypoplasia and MIH were detected in only five and two children, respectively. Because these sample sizes were extremely small, no statistical analysis was performed, and only descriptive information is presented. All affected children had deficient or insufficient vitamin D levels. Although this observation is broadly consistent with previous reports, the very small number of cases precludes any meaningful inference regarding a potential association between vitamin D status and these conditions.^13,27
^


This study has several limitations. It was a cross-sectional, single-centre study, which limits generalisability. Vitamin D levels may have been influenced by season, as measurements were taken during months with reduced sunlight. Because no questionnaire was used, it was not possible to adjust for sun exposure, dietary intake, supplementation, or seasonal patterns. In addition, some analyses were based on combined caries experience categories across different dentition stages, and age-adjusted analyses were not performed. Thus, the potential influence of age-related confounding cannot be completely excluded.

Further research with larger, multicentre samples, longitudinal follow-up, and comprehensive assessment of confounding factors is needed to clarify the relationship between vitamin D status and dental caries.

## Conclusion

Within the limitations of this cross-sectional study, several conclusions were drawn. No significant association was found between serum 25(OH)D levels and caries experience. A significant difference in serum 25(OH)D levels was observed between sexes, with girls having lower vitamin D levels than boys (46.03 ± 15.21 nmol/l vs. 54.63 ± 17.10 nmol/l, *P* < 0.001). Given the cross-sectional design and single-timepoint assessment of serum 25(OH)D levels, the present findings should be interpreted as associative rather than causal. Further longitudinal studies with comprehensive assessment of potential confounding factors are needed to better clarify the relationship between vitamin D status and dental caries.

### Acknowledgements

The authors would like to thank the staff of the Department of Pediatrics, Ordu University Training and Research Hospital, for their cooperation during data collection. The authors also thank all the children and their parents/legal guardians for their voluntary participation in this study. Permission for the study was obtained from Ordu University Clinical Research Ethics Committee with the decision number 2023/295. The study was conducted in accordance with the principles of the Declaration of Helsinki. Written informed consent was obtained from the legal guardians of all participants, and assent was also obtained from the children in accordance with ethical guidelines.

## References

## References

[ref1] Akman AO, Tumer L, Hasanoglu A, Ilhan M, Caycı B (2011). Frequency of vitamin D insufficiency in healthy children between 1 and 16 years of age in Turkey. Pediatr Int.

[ref2] Alhamed MS, Alharbi F, Al Joher A (2024). Vitamin D deficiency in children and adolescents in Saudi Arabia: A systematic review. Cureus.

[ref3] Altun C, Güven G, Başak F, Akbulut E (2005). Evaluation of children in the age group of 6 to 11 with respect to oral-dental health. Gulhane Med J.

[ref4] Anil S, Anand PS (2017). Early childhood caries: Prevalence, risk factors, and prevention. Front Pediatr.

[ref5] Bener A, Hoffmann G, Al Darwish M (2013). Vitamin D deficiency and risk of dental caries among young children: A public health problem. Indian J Oral Sci.

[ref6] Berdal A, Papagerakis P, Hotton D, Bailleul-Forestier I, Davideau JL (1995). Ameloblasts and odontoblasts, target-cells for 1,25-dihydroxyvitamin D3: A review. Int J Dev Biol.

[ref7] Bumbu BA, Luca MM, Buzatu R (2023). Examining the role of vitamin D in caries susceptibility in children’s deciduous teeth: A systematic review. Nutrients.

[ref8] Buzatu R, Luca MM, Bumbu BA (2024). A systematic review of the relationship between serum vitamin D levels and caries in the permanent teeth of children and adolescents. Dent J (Basel).

[ref9] Carvalho Silva C, Gavinha S, Manso MC (2021). Serum levels of vitamin D and dental caries in 7-year-old children in Porto metropolitan area. Nutrients.

[ref10] Doğusal G, Sönmez I, Ünüvar T (2021). Evaluation of serum 25(OH)D levels in obese and normal-weight children with carious and hypomineralized teeth. J Clin Pediatr Dent.

[ref11] Dudding T, Thomas SJ, Duncan K, Lawlor DA, Timpson NJ (2015). Re-examining the association between vitamin D and childhood caries. PLoS One.

[ref12] Duman S, Bilmez Selen M, Demir P (2022). Evaluation of the relationship between severe early childhood caries and vitamin D. Pediatr Dent J.

[ref13] Fulton A, Amlani M, Parekh S (2020). Oral manifestations of vitamin D deficiency in children. Br Dent J.

[ref14] Güler Ç, Eltas A, Güneş D, Görgen VA, Ersöz M (2012). Malatya İlindeki 7-14 Yaş Arası Çocukların Ağız-Diş Sağlığının Değerlendirilmesi. Ann Health Sci Res.

[ref15] Gyll J, Ridell K, Öhlund I, Karlsland Åkeson P, Johansson I, Lif Holgerson P (2018). Vitamin D status and dental caries in healthy Swedish children. Nutr J.

[ref16] Hatun S, Islam Ö, Cizmecioglu F (2005). Subclinical vitamin D deficiency is increased in adolescent girls who wear concealing clothing. J Nutr.

[ref17] Hocaoğlu-Emre FS, Sarıbal D, Oğuz O (2019). Vitamin D deficiency and insufficiency according to the current criteria for children: Vitamin D status of elementary school children in Turkey. J Clin Res Pediatr Endocrinol.

[ref18] Holick MF (2007). Vitamin D deficiency. N Engl J Med.

[ref19] Holick MF, Binkley NC, Bischoff-Ferrari HA (2011). Evaluation, treatment, and prevention of vitamin D deficiency: An Endocrine Society clinical practice guideline. J Clin Endocrinol Metab.

[ref20] Hooley M, Skouteris H, Boganin C, Satur J, Kilpatrick N (2012). Parental influence and the development of dental caries in children aged 0–6 years: A systematic review of the literature. J Dent.

[ref21] Isa H, Almaliki M, Alsabea A, Mohamed A (2020). Vitamin D deficiency in healthy children in Bahrain: Do gender and age matter. East Mediterr Health J.

[ref22] Kale S, Kakodkar P, Shetiya S, Abdulkader R (2020). Prevalence of dental caries among children aged 5-15 years from 9 countries in the Eastern Mediterranean Region: A meta-analysis. East Mediterr Health J.

[ref23] Karaca A, Altın H (2024). Vitamin D status in children in the South Marmara Region in Turkey. J Contemp Med.

[ref24] Karagüzel G, Dilber B, Çan G, Ökten A, Değer O, Holick MF (2014). Seasonal vitamin D status of healthy schoolchildren and predictors of low vitamin D status. J Pediatr Gastroenterol Nutr.

[ref25] Kennel KA, Drake MT, Hurley DL (2010). Vitamin D deficiency in adults: When to test and how to treat. Mayo Clin Proc.

[ref26] Kragt L, van der Tas JT, Moll HA (2016). Early caries predicts low oral health-related quality of life at a later age. Caries Res.

[ref27] Kühnisch J, Thiering E, Kratzsch J (2015). Elevated serum 25(OH)-vitamin D levels are negatively correlated with molar-incisor hypomineralization. J Dent Res.

[ref28] Li Z, Wei X, Shao Z, Liu H, Bai S (2023). Correlation between vitamin D levels in serum and the risk of dental caries in children: a systematic review and meta-analysis. BMC Oral Health.

[ref29] Manios Y, Moschonis G, Hulshof T (2017). Prevalence of vitamin D deficiency and insufficiency among schoolchildren in Greece: The role of sex, degree of urbanisation and seasonality. Br J Nutr.

[ref30] Mansbach JM, Ginde AA, Camargo CA (2009). Serum 25-hydroxyvitamin D levels among US children aged 1 to 11 years: Do children need more vitamin D. Pediatrics.

[ref31] Navarro CLA, Grgic O, Trajanoska K (2021). Associations between prenatal, perinatal, and early childhood vitamin D status and risk of dental caries at 6 years. J Nutr.

[ref32] Öztunç H, Haytaç MC, Özmeriç N, Uzel İ (2000). Adana ilinde 6-11 yaş grubu çocukların ağız-diş sağlığı durumlarının değerlendirilmesi. Gazi Üniv Diş Hek Fak Derg.

[ref33] Qin X, Wang M, Wang L, Xu Y, Xiong S (2024). Association of vitamin D receptor gene polymorphisms with caries risk in children: A systematic review and meta-analysis. BMC Pediatr.

[ref34] Roh YE, Kim BR, Choi WB (2016). Vitamin D deficiency in children aged 6 to 12 years: Single center’s experience in Busan. Ann Pediatr Endocrinol Metab.

[ref35] Schroth RJ, Rabbani R, Loewen G, Moffatt ME (2016). Vitamin D and dental caries in children. J Dent Res.

[ref36] Selwitz RH, Ismail AI, Pitts NB (2007). Dental caries. Lancet.

[ref37] Seminario AL, Velan E (2016). Vitamin D and dental caries in primary dentition. J Dent Child (Chic).

[ref38] Unger MD, Cuppari L, Titan SM (2010). Vitamin D status in a sunny country: Where has the sun gone. Clin Nutr.

[ref39] van der Tas JT, Kragt L, Elfrink MEC (2017). Social inequalities and dental caries in six-year-old children from the Netherlands. J Dent.

[ref40] Vargas-Ferreira F, Salas MMS, Nascimento GG (2015). Association between developmental defects of enamel and dental caries: A systematic review and meta-analysis. J Dent.

[ref41] Wacker M, Holick M (2013). Vitamin D — Effects on skeletal and extraskeletal health and the need for supplementation. Nutrients.

[ref42] Wetzel WE, Reckel U (1991). Defective 6-year molars increasing--An inquiry. Zahnarztl Mitt.

[ref44] Zemaitiene M, Grigalauskiene R, Andruskeviciene V (2017). Dental caries risk indicators in early childhood and their association with caries polarization in adolescence: A cross-sectional study. BMC Oral Health.

[ref45] Zhang Z, Qiu S, Wang Z, Hu Y (2024). Vitamin D levels and five cardiovascular diseases: A Mendelian randomization study. Heliyon.

